# Mer receptor tyrosine kinase is frequently overexpressed in human non-small cell lung cancer, confirming resistance to erlotinib

**DOI:** 10.18632/oncotarget.3280

**Published:** 2015-03-16

**Authors:** Shengzhi Xie, Yongwu Li, Xiaoyan Li, Linxiong Wang, Na Yang, Yadi Wang, Huafeng Wei

**Affiliations:** ^1^ International Joint Cancer Institute, Second Military Medical University, Shanghai, China; ^2^ Department of Oncology, General Hospital of Chinese People's Armed Police Forces, Beijing, China; ^3^ Department of Radiology, 302 Hospital of Chinese People's Liberation Army, Beijing, China; ^4^ Department of Lung Cancer, Affiliated Hospital of Academy of Military Medical Sciences, Beijing, China; ^5^ Cancer Center Lab, Chinese People's Liberation Army General Hospital & Beijing Key Laboratory of Cell Engineering & Antibody, Beijing, China; ^6^ South Building No. 2 Division, General Hospital of Chinese People's Armed Police Forces, Beijing, China; ^7^ Department of Radiation Oncology, General Hospital of Beijing Military Region, Beijing, China

**Keywords:** mer receptor tyrosine kinase, NSCLC, targeted therapy, erlotinib, resistance

## Abstract

Mer is a receptor tyrosine kinase (RTK) with oncogenic properties that is often overexpressed or activated in various malignancies. Using both immunohistochemistry and microarray analyses, we demonstrated that Mer was overexpressed in both tumoral and stromal compartments of about 70% of non-small cell lung cancer (NSCLC) samples relative to surrounding normal lung tissue. This was validated in freshly harvested NSCLC samples; however, no associations were found between Mer expression and patient features. Although Mer overexpression did not render normal lung epithelial cell tumorigenic *in vivo*, it promoted the *in vitro* cell proliferation, clonogenic colony formation and migration of normal lung epithelial cells as well as NSCLC cells primarily depending on MAPK and FAK signaling, respectively. Importantly, Mer overexpression induced resistance to erlotinib (EGFR inhibitor) in otherwise erlotinib-sensitive cells. Furthermore, Mer-specific inhibitor rendered erlotinib-resistant cells sensitive to erlotinib. We conclude that Mer enhances malignant phenotype and pharmacological inhibition of Mer overcomes resistance of NSCLC to EGFR-targeted agents.

## INTRODUCTION

Lung cancer is the leading cause of cancer-related death worldwide, causing about 1·6 million deaths in 2012 where about 40% of cases occurs in China [1]. Non-small cell lung cancer (NSCLC) accounts for 80–85% of lung cancer cases and its current 5-year survival rate for all stages of disease is only 17% [2]. Last decade has witnessed the discovery of molecular mutations that drive lung cancer in a substantial minority of patients and development of many targeted therapeutics that have significantly improved outcome in those patients. The well-characterized molecular changes involve the driver mutations in the genes of *EGFR* (~10%), *ALK* (5%), *KRAS* (10–20%), *PI3KCA* (3%), *BRAF* (3%), *ROS1* (1%), *c-MET* (2%) and *ERBB2* (2%) etc [3]. Therapeutic agents targeting these molecular aberrations in cancer cells have been effective at prolonging survival of patients [4]; however, for the remaining majority of patients with NSCLC, the oncogenic drivers are complex and identification of additional therapeutic targets has become a major research focus [5]. To address this problem, we have investigated the roles of Mer receptor tyrosine kinase (RTK) as a novel oncogenic molecule in lung cancer.

Mer RTK belongs to the Tyro3, Axl, and Mer (TAM) family of RTKs [6, 7]. Abnormal activation of the TAM receptors is implicated in the oncogenesis of a spectrum of human cancers, including hematological malignancies and glioblastoma, melanoma, prostate cancer, breast cancer, colon cancer, gastric cancer, pituitary adenomas, and rhabdomyosarcomas [8]. Previous studies identified Axl as a potential therapeutic target in NSCLC, particularly in adenocarcinoma, where Axl expression correlated with tumor progression, malignant behavior of tumor cells, and tumor resistance to chemo- and targeted therapies [9–13]. With regard to Mer, a recent study demonstrates that Mer RTK is overexpressed in about 70% of NSCLC relative to surrounding normal lung tissue where Mer functions to enhance the proliferation of cancer cells and inhibits their apoptosis, thereby promoting chemoresistance [5]; moreover, knockdown of Mer expression by short hairpin RNA (shRNA) abrogated oncogenic phenotypes of tumor cells, including decreased clonogenic growth, improved chemosensitivity, and delayed tumor progression in animal models [5], thus identifying it as a potential therapeutic target in NSCLC [14].

However, the above study of Mer expression was conducted in a relatively small cohort of NSCLC samples [5]; though the downstream signaling pathways of Mer activation have been dissected, further knowledge of deeper mechanisms for Mer-mediated oncogenic phenotypes remains needed. In addition, macrophages have been described constitutively express Mer receptor by which they constantly phagocytose apoptotic cells to maintain self-tolerance in the steady state [15], and immunosuppressive agents have been demonstrated be able to further upregulate the expression of Mer [16]. In view of the abundant presence of tumor-associated macrophages and immunosuppressive factors in tumors [17], it would be interesting to explore the expression and its clinical significance of stromal Mer in tumors. Therefore, in the present study, we first examined the Mer expression in both tumoral and stromal compartments by using tissue microarrays (TMA) containing a relatively large amount of NSCLC samples (150 cases) and repeated the findings in freshly harvested NSCLC samples (30 cases) by using immunohistochemistry and western blotting, and then correlated the findings with clinicopathological features of patients. We further explored the biological effects of Mer expression in lung epithelial cells and NSCLC cells by using both overexpression and function-blocking experiments.

## RESULTS

### Mer is frequently overexpressed and activated in NSCLC

**Figure 1:**
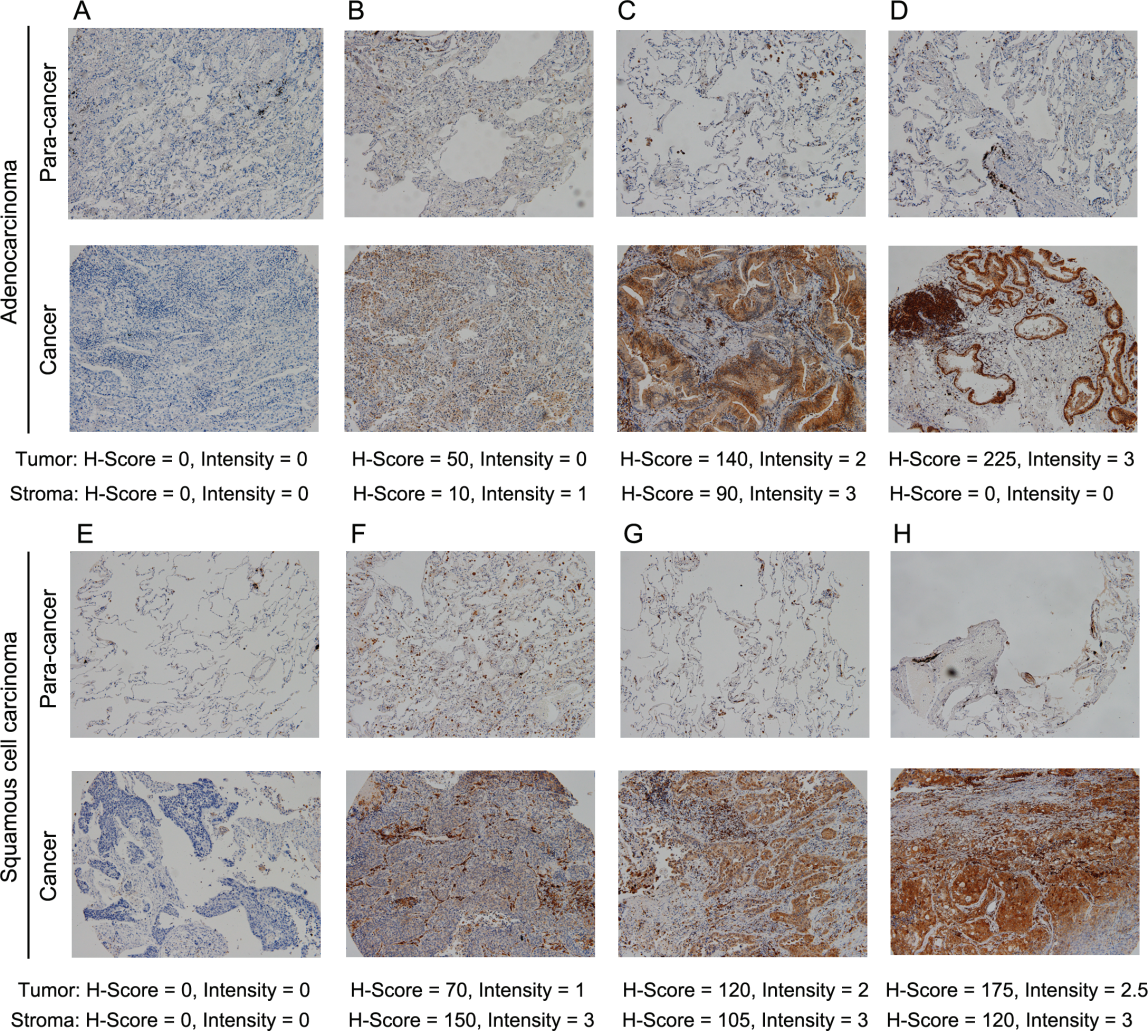
Mer RTK is frequently expressed in NSCLC Immunohistochemical staining of Mer in sections of adenocarcinoma (**(A-D)**, bottom panels) and squamous cell carcinoma of human lung (**(E-H)**, bottom panels) and matched paracancerous tissues (A-H, upper panels). The staining intensity of Mer increases from left to right pictures as indicated by H-score below corresponding graph. All representative pictures were shown in 100 × magnification.

We first evaluated expression of Mer in TMA containing cancer tissues with matched paracancerous tissues from 150 Chinese patients with NSCLC. Demographic and histopathological data are presented in Table 1. Concordant with previous reports, survival was associated with age and stage of disease (TNM stage and lymph node status), but not histological subtype and differentiation degree [5, 18]. Tumor cells exhibited membranous and cytoplasmic staining for Mer (Fig. 1A–1H, lower panels). The staining was specific since no staining was noted when PBS was used instead of primary anti-Mer antibody (Supplementary Fig. 1A). Mer expression in tumor cells (MERt) was detected (H-score ≥ 5) in 67% of patients (Table 1) and was generally low-to-moderate with a median H-score of 10 (range: 0–300) while intermediate (H-score = 101–200) and high (H-score = 201–300) Mer expression was seen in 11% and 2% of patients respectively. Within the tumor microenvironment, Mer was strongly expressed in cells exhibiting macrophage morphology, but not in blood vessels (Fig. 1A–1H, lower panels and Supplementary Fig. 1B and 1C). Stromal Mer expression (MERs) was observed in 73% of patients (Table 1) and was also low-to-moderate with a median H-score of 15 while intermediate and high Mer expression in tumor stroma was found in 7% and 3% of patients respectively. Normal lung epithelial tissue adjacent to tumors was always negative (Fig. 1A–1H, upper panels) while stromal cells with macrophage morphology sometimes show positive for Mer (Fig. 1C and 1F, upper panels), indicating up-regulated Mer expression in both cancer cells and stromal cells. We also found the evident correlation between tumoral and stromal Mer expression (*r* = 0.4784, *p* < 0.0001, Supplementary Fig. 2).

**Table 1:** Associations between molecular/clinical features and overall survival in 150 NSCLC patients (univariate analysis; log-rank test)

	*Patients* N (%)	*p*	*Survival at the 50th percentile*	
			*Estimate*	*95% CI*
*Age at diagnosis*				
≤ 63 years	77 (51)	< 0.001	63	60–66
> 63 years	73 (49)		49	35–63
*Gender*				
Male	109 (73)	0.942	59	55–62
Female	41 (27)		64	57–71
*Histology*				
Adeno	66 (44)	0.879	61	56–65
SCC	75 (50)		59	54–64
BAC	9 (6)		61	55–67
*Stage*				
I	61 (41)		63	59–67
II	28 (19)		58	40–76
III	47 (31)	< 0.001	50	26–74
IV	4 (3)		41	12–69
UNK	10 (6)		49	29–69
*Nodal status*				
0	80 (53)		63	59–66
1	24 (16)	< 0.001	38	8–68
2–3	41 (27)		48	32–64
UNK	5 (4)		64	61–66
*Differentiation*				
Poor	12 (8)		57	50–64
Moderate	95 (63)	0.743	61	57–65
Well	41 (27)		58	44–72
UNK	2 (1)		66	NR
*MERt H-score*				
0–4	50 (33)		57	48–67
5–100	81 (54)	0.337	60	56–64
101–200	16 (11)		63	47–78
201–300	3 (2)		90	18–162
*MERs H-score*				
0–4	41 (27)		58	53–62
5–100	95 (63)	0.792	61	56–65
101–200	10 (7)		44	1–87
201–300	4 (3)		89	86–91

Abbreviations: Adeno, adenocarcinoma; BAC, bronchioalveolar carcinoma; SCC, squamous cell carcinoma; UNK, unknown; NR, not reached; MERt, MER in tumor; MERs, MER in stroma; Survival time (month) was estimated at the 50th percentiles.

**Figure 2:**
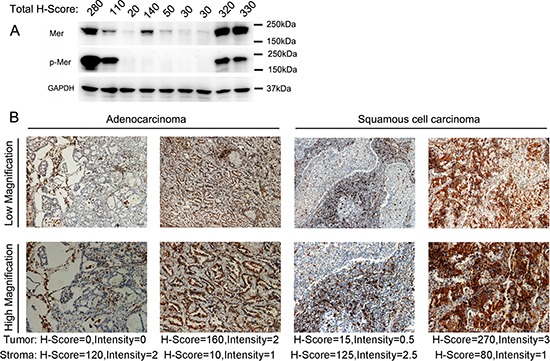
Overexpression of Mer RTK in NSCLC of freshly harvested cancer tissues **(A)** Evaluation of Mer expression and activation (phosphorylated Mer, p-Mer) in lysates of 9 freshly harvested NSCLC tissues by western blotting. The corresponding H-score of each sample evaluated by IHC is listed on the top of each lane. **(B)** Immunohistochemical staining of Mer in sections of freshly harvested NSCLC tissues with representative pictures of adenocarcinoma (left) and squamous cell carcinoma (right). The scale bars in the pictures of low and high magnification represent 50 μM and 100 μM respectively.

To further confirm the Mer expression, we harvested fresh neoplastic tissues from 30 patients with NSCLC and subjected them to analysis by IHC and western blotting. Consistent with the results from TMA, Mer expression was detected (H-score ≥ 5) in both tumoral and stromal compartments of about 70% of patients with predominant low (H-score = 5–100) and intermediate (H-score = 101–200) levels (Supplementary Table 1). The representative pictures were shown in Fig. 2B. In addition, we detected the Mer expression in all 10 samples selected from 30 patients by western blot analysis with expression level similar to that seen in IHC (Fig. 2A). The 4 samples with intermediate and high Mer expression also exhibit Mer phosphorylation (pMer) in the western blots, indicating Mer activation in these samples. These data are consistent with and expand on previous reports of elevated Mer protein expression in NSCLC [5, 19], and represent the first report of Mer overexpression in both tumoral and stromal compartments of these tumors.

### No correlation between Mer expression and clinical features

**Figure 3:**
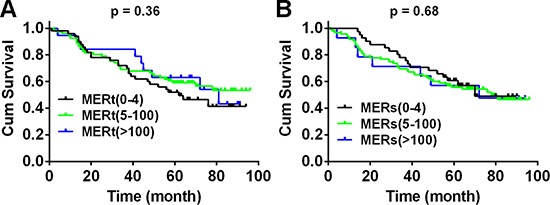
Mer expression is not correlated with overall survival of patients with NSCLC Kaplan–Meier curves for overall survival of 150 NSLCC patients according to the Mer expression level in tumoral and stromal compartments over a median 60.5-month follow up period after initial surgery. Due to few cases with H-score of Mer at 101–200 and 201–300, we integrated these two groups into one group (Mer > 100). The results for unintegrated groups were shown in Supplementary Fig. 3A.

We next dissected the correlations between Mer expression and clinical characteristics from TMA-contained samples. Mer expression in both tumoral and stromal compartments has no associations with overall survival of patients (Fig. 3 and Supplementary Fig. 3A) and did not differ significantly by TNM stage, histology and differentiation (Supplementary Fig. 3B). We also did not found any correlation between Mer expression and clinical features in 30 samples from freshly harvested tissues (data not shown).

### Mer expression promotes the proliferation and migration in lung epithelial cells

**Figure 4:**
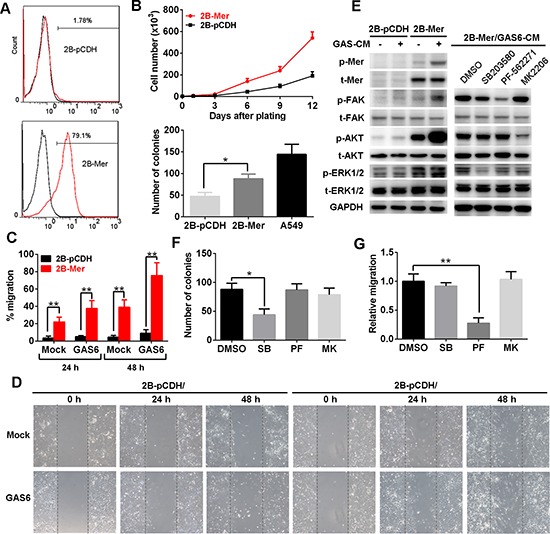
Mer overexpression promotes the proliferation and migration of normal lung epithelial cells **(A)** Mer expression in BEAS2B cells stably transfected with control (2B-pCDH) or Mer-carried lentivirus (2B-Mer) determined by flow cytometry. **(B)** Proliferation and clonogenic colony formation assays. BEAS2B cells stably expressing control (2B-pCDH) or Mer (2B-Mer) were plated in 6-well plates and cultured for 12 days or 10 days, and cell counts were performed every 3days (upper) or colony numbers were determined at the end of experiments (bottom). **(C and D)** Migration assays. BEAS2B cells stably expressing control (2B-pCDH) or Mer (2B-Mer) were plated on 6-well plates where wounds were made using a pipette tip and cell migration into wounds were monitored at 24 and 48 h after wounding in the presence of 20% (v/v) mock or GAS6 contained CM. The representative photographs taken immediately (0 h) and 24 and 48 h after wounding were shown in D. **(E)** Left, the activation of Mer and its downstream signals was determined by western blotting using antibodies specific for total (t) and phosphorylated (p) proteins in BEAS2B cells stably expressing control (2B-pCDH) or Mer (2B-Mer) cultured in the presence of 20% (v/v) mock or GAS6 contained CM. Right, blockade of signaling pathway activity downstream of Mer activation in 2B-Mer cells treated with the corresponding signal inhibitors. Blots representative of three independent experiments were shown. **(F)** Clonogenic colony formation from 2B-Mer cells cultured in the presence of the corresponding inhibitors for MAPK (SB203580, 1 μM), FAK (PF-562271, 1 μM)) and AKT (MK2206, 4 μM)) signals with DMSO treatment as controls. **(G)** Relative cell migration of 2B-Mer cells 48 h after wounding in the presence of the corresponding inhibitors for AKT, MAPK and FAK signals with DMSO treatment as controls. All data are expressed as mean ± SD of triplicates and representative of three independent experiments. *p < 0.05, **p < 0.01, paired student *t* test (for C) or ANOVA followed by Tukey's multiple comparisons test (for B, F and G).

To dissect the biological role of Mer expression in lung cancer initiation, we performed the study on the biological effects of Mer overexpression in lung epithelial cells. We first stably expressed Mer using lentiviral transduction in BEAS2B cells, an immortalized human bronchial epithelial cell lines devoid of endogenous Mer (Fig. 4A). As shown in Fig. 4B, Mer expression on the cell surface of BEAS2B cells promoted the *in vitro* cell proliferation and colony formation. The BEAS2B cells are not tumorigenic in nude mice, and *in vivo* injection of BEAS2B-Mer cells did not form tumor nodules while BEAS2B cells stably expressing EML4-ALK, a well-known transforming oncoprotein [20], did form subcutaneous xenografts (Supplementary Table 2), indicating a lack of transforming activity for Mer. Furthermore, BEAS2B-Mer cells were found to have significantly increased migration compared with control BEAS2B-pCDH cells, which further enhanced by the addition of GAS6-contained conditioned media (Fig. 4C and 4D). Consistent with previous study, we observed that Mer expression stimulates MAPK (ERK1/2), AKT and FAK signaling pathways in BEAS2B cells which can be further potentiated by the addition of GAS6-contained conditioned media (Fig. 4E, left panel). To dissect the key signaling pathways responsible for the increased proliferation and migration of Mer-expressing BEAS-2B cells, we subjected them to the treatments with the inhibitors specific for signaling pathways activated by Mer (Fig. 4E, right panel). The result showed that inhibition of MAPK pathway by SB203580 and FAK pathway by PF-562271 significantly blocked the increased proliferation and migration of Mer-expressing BEAS-2B cells respectively (Fig. 4F and 4G), indicating a critical role of MAPK and FAK activation in mediating Mer-elicited proliferation and migration in lung epithelial cells respectively. As a control, we saw few effects of pathway inhibitors on cell proliferation and migration in control BEAS2B-pCDH cells (Supplementary Fig. 4, left panels).

To substantiate the above findings in lung epithelial cells, we conducted the similar experiments in PC9 NSCLC cell line which itself exhibits litter, if any, expression of endogenous Mer (Supplementary Fig. 5A). Likewise, we observed that Mer overexpression promoted the *in vitro* colony formation and migration in PC9 cells (Supplementary Fig. 5B–5D). The increased proliferation and migration of PC9 cells expressing Mer were dependent on MAPK and FAK signaling pathways downstream of Mer activation in that blockade of these two pathways abrogated the increased proliferation and migration of PC9-Mer cells respectively (Supplementary Fig. 5E–5G). These two pathway inhibitors had a mild effect in control PC9-pCDH cells, which was far from reaching the suppressive effects seen in PC9-MER cells (Supplementary Fig. 4, right panels).

**Figure 5:**
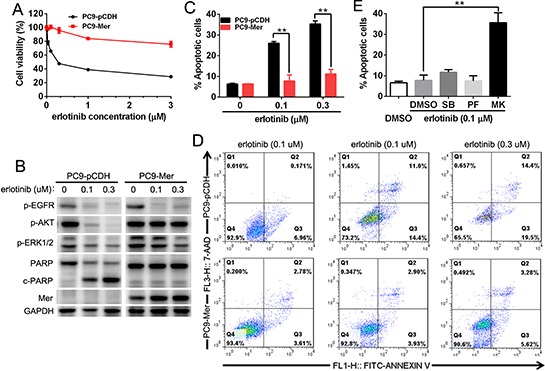
Mer overexpression induces the resistance of NSCLC cells to erlotinib **(A)** PC9 cells stably expressing control (PC9-pCDH) or Mer (PC9-Mer) were treated with serially diluted concentrations of erlotinib for 72 h and cell viability was determined by MTT assay. **(B)** PC9 cells stably expressing control (PC9-pCDH) or Mer (PC9-Mer) were treated with erlotinib at indicated concentrations for 24 h and then activation of EGFR-related signaling pathway and PARP cleavage were evaluated by western blotting. Blots representative of three independent experiments were shown. **(C and D)** PC9 cells stably expressing control (PC9-pCDH) or Mer (PC9-Mer) were treated with erlotinib at indicated concentrations for 24 h and then cell apoptosis were determined by Annexin V/7AAD staining. The representative dotplots of three experiments were shown in D. **(E)** PC9 cells stably expressing Mer (PC9-Mer) were pretreated with the corresponding inhibitors for MAPK (SB203580, 1 μM), FAK (PF-562271, 1 μM)) and AKT (MK2206, 4 μM)) signals with DMSO treatment as controls for 4 h followed by addition of erlotinib for 20 h and cell apoptosis were determined by Annexin V/7AAD staining. All data are expressed as mean ± SD of triplicates and representative of three independent experiments. **p < 0.01, paired student *t* test (for C) or ANOVA followed by Tukey's multiple comparisons test (for E).

### Mer expression induces the resistance of NSCLC cells to erlotinib treatment

Previous study shows that Mer knockdown improves *in vitro* sensitivity of NSCLC cells to chemotherapeutic drugs by increased apoptosis induction, however, whether it is the case for EGFR inhibitor remains unexplored. To do this, we determined the sensitivity of EGFR mutation-harbored PC9 cells (delE746-A750) with or without stable Mer overexpression to EGFR inhibitor erlotinib by MTT assay. Mer overexpression induced a notable resistance to erlotinib treatment in otherwise elrotinib-sensitive PC9 cells, resulting in an approximate 24-fold increase in the half-maximal inhibitory concentrations (IC50) of erlotinib (0.34 μM vs 8.22 μM for control or Mer overexpression; Fig. 5A). Analysis of EGFR-related signaling pathways demonstrated that erlotinib treatment did not block the AKT phosphorylation in PC9-Mer cells though it inhibited the EGFR and MAPK phosphorylation in both PC9-pCDH and PC9-Mer cells (Fig. 5B). We further applied Annexin V/7-AAD staining to analyze the apoptosis of PC9-pCDH and PC9-Mer cells in response to erlotinib treatment. PC9-Mer cells exhibited a significant decrease in apoptosis relative to control PC9-pCDH cells following erlotinib treatment (Fig. 5C). The representative dotplots were shown in Fig. 5D. In addition, compared with control PC9-pCDH cells, PC9-Mer cells displayed a significantly decreased PARP cleavage in response to erlotinib treatment indicative of decreased apoptotic pathway activation at the biochemical level (Fig. 5B). Furthermore, blockade of AKT signaling pathway by MK2206 recovered the sensitivity of PC9-Mer cells to erlotinib treatment (Fig. 5E), suggesting that Mer expression could be an advantage for cancer cells by enhancing drug resistance through sustained AKT activation.

### Mer inhibition reversed the resistance of NSCLC cells to erlotinib treatment

**Figure 6:**
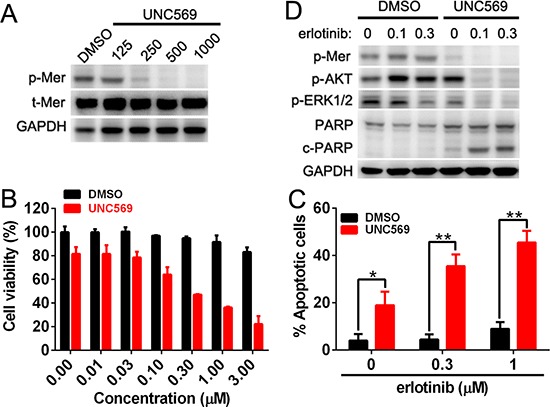
Mer inhibition rescues the sensitivity of NSCLC cells to erlotinib treatment **(A)** H1965 cells were treated with Mer-specific inhibitor UNC569 at indicated concentrations for 12 h and Mer phosphorylation was determined by western blotting. **(B)** H1965 cells were pretreated with Mer-specific inhibitor UNC569 or vehicle control (DMSO) for 12 h followed by incubation with serially diluted concentrations of erlotinib for 72 h, and cell viability was evaluated by MTT assay. All data were normalized to the control of DMSO treatment. (C and D) H1965 cells were pretreated with Mer-specific inhibitor UNC569 or vehicle control (DMSO) for 12 h followed by incubation with erlotinib at indicated concentrations for 24 h. Cell apoptosis was determined by Annexin V/7AAD staining **(C)** and Mer-related signaling pathway and PARP cleavage were evaluated by western blotting **(D)** Blots representative of three independent experiments were shown. All data are expressed as mean ± SD of triplicates and representative of three independent experiments. *p < 0.05, **p < 0.01, paired student *t* test (for C).

To define the role of endogenous Mer expression in dictating the sensitivity of NSCLC cells to EGFR inhibitor, we selected H1965 cells harboring EGFR mutation (delE746-A750) which is insensitive to erlotinib treatment and express the high level of endogenous Mer. We pretreated H1965 cells with UNC569, a recently developed small-molecule tyrosine kinase inhibitor specific for Mer, to block the activation of Mer, and then evaluated the sensitivity of these cells to erlotinib treatment by MTT assays. As shown in Fig. 6A, treatment with 500 nmol/L of UNC569 effectively inhibited the phosphorylation of Mer; furthermore, pretreatment of H1965 cells with UNC569 at this concentration significantly improved the sensitivity of H1965 cells to erlotinib, resulting in an approximate 50-fold reductions in the IC50 of erlotinib (13.65 μM vs 0.28 μM for DMSO or UNC569 pretreatment; Fig. 6B). Annexin V/7-AAD staining demonstrated that UNC569-pretreated H1965 cells exhibited a significantly increased apoptosis relative to control pretreatment in response to elrotinib treatment (Fig. 6C), which was also confirmed by increased production of cleaved PARP at the biochemical level (Fig. 6D). Consistent with previous studies [5], we also observed that UNC569 treatment alone decreased H1965 cell viability by inducing their apoptosis (Fig. 6B–6D), suggesting a role of Mer in the survival of Mer-overexpressed NSCLC cells. Treatment with UNC569 and erlotinib simultaneously inhibited the phosphorylation of MAPK and AKT whereas either erlotinib or UNC569 treatment alone merely inhibited the activation of MAPK but not AKT signaling pathway, which at least partially explained the synergistic pro-apoptotic effect of both UNC569 and erlotinib in H1965 cells (Fig. 6D) and was also consistent with above results obtained in PC9 cells (Fig. 5F). The data indicate that abrogating the function of endogenous Mer can improve the therapeutic efficacy of elrotinib treatment in otherwise erlotinib-insensitive NSCLC cells harboring EGFR mutation.

## DISCUSSION

Though the biological functions of Mer receptor have long been well characterized in many cancers, it is now that we start to be aware of the significance of Mer receptor in the tumorigenesis of lung cancer. This paper extends the findings of a previous report that has identified Mer receptor frequently overexpressed in human NSCLC relative to surrounding normal lung tissue [5]; it further shows that Mer receptor is short of transforming activity since it did not confer normal lung epithelial cells tumorigenic activity when overexpressed in them, though it did markedly promote their proliferation and migration. More importantly, Mer expression dictates the response to EGFR-targeted small-molecule inhibitor of NSCLC cells with *EGFR* gene mutation, warranting further investigation of Mer inhibitors as potential therapeutic agents in NSCLC.

This study shows Mer overexpression in tumor cells of about 70% NSCLC samples of TMA and freshly harvested tissues compared to matched paracancerous tissues; moreover, Mer phosphorylation was also observed among freshly harvested tissue samples with high level of Mer expression. Gas6, protein S and galectin-3, the ligands for Mer, have been also described to be frequently overexpressed in tumor tissues of NSCLC [21–23], suggesting that Mer receptor is continuously activated in NSCLC via autocrine and/or paracrine mechanisms. Consistent with the previous findings [5], we also did not find that Mer expression is associated with any clinical feature of patients with NSCLC, including overall survival of patients. It is more likely that the level of Mer phosphorylation other than total Mer expression is related with clinical parameters of patients; thus, future studies should evaluate the phosphorylation status of Mer in NSCLC tumor tissues to investigate whether Mer activation frequently occurs and whether it has prognostic or predictive value.

Previous studies have demonstrated that Mer expression promotes the growth and survival of NSCLC cells [5], however, the biological effects of Mer expression in normal lung epithelial cells remain unexplored. Herein, we first found that Mer overexpression in human bronchial epithelial cell line BEAS2B similarly enhanced the proliferation, colony formation and migration, but did not confer them of tumorigenic activity *in vivo*, indicating a shortage of transforming activity for Mer in lung epithelial cells. The results argue that, unlike the role in leukemogenesis [24, 25], Mer expression is more likely a propagating (passenger) factor than a driver factor in the oncogenesis of NSCLC, which is also supported by few of activating mutation of Mer in NSCLC and other cancers [26, 27]. It is very likely that preneoplastic cells harboring driver mutation get an additional impetus to progress after Mer overexpression, which is also consistent with the conception that TAM RTKs have a differential role in a cell type-specific context [28]. It remains to learn when and how upregulated Mer expression occurs in the tumorigenesis of NSCLC; the fact that Mer expression was not correlated with tumor stage suggests that *de novo* expression of Mer in tumor cells occurs at the early stage of tumorigenesis. Previous studies demonstrate that Axl, another member of TAM RTK, is a transcriptional target of both wild-type and mutant p53 [29], suggesting that Axl can be induced by a driver of tumorigenesis; thus it is attempting to speculate that mutant drivers of p53 etc. might likewise upregulate the expression of Mer in the mutant p53-carried NSCLC, which is initially supported by the online Whole Genome RVista analysis showing the presence of 12 p53 binding sites in the promoter region of *Mer* gene (Supplementary Fig. 5). Future experiment studies are needed to validate the above hypothesis and to see if Mer expression is positively correlated with p53 expression in tumor tissues. In addition, many NSCLC lose p53 entirely leading to the unavailability of p53 to promote Mer expression [30], therefore, other unappreciated mechanisms for Mer overexpression would be operative in these tumors which remains to be explored.

Previous studies show that Mer activation stimulated MAPK, AKT and FAK signaling pathways and also a novel pro-survival pathway involving AKT, CREB, Bcl-xL, survivin, and Bcl-2 [5]. This study advanced previous findings and defined that increased proliferation and migration conferred by Mer overexpression are primarily dependent on MAPK and FAK signaling pathways, respectively as blockade of MAPK and FAK signaling pathways abrogated the increased proliferation and migration in Mer-overexpressed lung epithelial cells and NSCLC cells, respectively. The similar mechanisms of action have also been identified for Mer in promoting proliferation and migration of tumor cells in other cancers, including melanoma, prostate cancer and glioblastoma etc [26, 31–34]. In addition, Axl has been shown to mediate migration and invasion of glioblastoma, lung cancer and breast cancer cells by upregulating the expression of matrix metalloproteinases and promoting the transition of solid tumor cells from an epithelial to mesenchymal morphology [9, 35–38], and whether Mer functions by similar mechanisms in NSCLC remains to be investigated.

More importantly, this study is the first report to document a key role for Mer in dictating the sensitivity of NSCLC cells to EGFR-targeted agent erlotinib. Our data clearly demonstrated that Mer overexpression significantly attenuated the sensitivity of PC9 cells to erlotinib treatment by decreasing apoptosis induction. Mechanistic investigation revealed that erlotinib treatment did not effectively inhibited AKT signaling in otherwise erlotinib-sensitive NSCLC cells upon Mer overexpression, suggesting that sustained AKT signal downstream of Mer activation mediated the erlotinib resistance as evidenced by recovered erlotinib sensitivity in Mer-overexpressed PC9 cells when concomitantly treated with an AKT inhibitor. By using Mer-specific inhibitor, we demonstrated that blockade of Mer activity increased the sensitivity of H1965 cells to erlotinib treatment, further validating the key role of endogenous Mer in the resistance of NSCLC cells to erlotinib. Previous studies showed that Mer was required for surface accumulation of EGFR and subsequent pathway activation in lung cancer cells [39]; thus, it is tempting to speculate that Mer might maintain the persistent activation of the key prosurvival PI3K/AKT signaling pathway in NSCLC cells with EGFR mutation exposed to erlotinib by the direct (activating AKT signal) and/or indirect (sustaining EGFR signal) mechanisms, convergently leading to the resistance of NSCLC cells to erlotinib. As persistent activation of PI3K/AKT pathway has been documented play a central role in mediating primary and/or acquired resistance of NSCLC harboring EGFR mutation to EGFR-targeted agents [40], the findings in this manuscript thus provide an alternative upstream source. Future studies are warranted to explore whether Mer expression and/or activation status in tumor tissues would be correlated with the response of patients with NSCLC harboring EGFR mutation to erlotinib or other EGFR-targeted agents.

Intriguingly, this study first shows that Mer immunopositivity is similarly present in stromal compartments of NSCLC samples which are predominantly found in cells with macrophage morphology, most likely tumor-associated macrophages. The Mer expression in non-neoplastic cells found in the tumor microenvironment may be of great significance; in the physiological steady states, one of major functions for Mer receptor expressed on the phagocytes of DCs and macrophages is responsible for the maintenance of tissue homeostasis and immune tolerance through efferocytosis [15], the physiological process by which apoptotic cells are engulfed by phagocytes [41]; in rapidly proliferating tumors, apoptosis occurs with a generally higher rate as compared with quiescent tissues; Mer receptor on tumor-associated macrophages may perform the same task of apoptotic cell clearance by which tumor-associated macrophages persistently uptake and present self-antigens thus enabling to suppress the induction of antitumor immunity. This hypothesis has been supported by the findings that Mer deficiency in CD11b^+^ leucocytes promoted a CD8^+^ T lymphocyte mediated antitumor immune response and inhibited tumor growth and metastasis in the murine tumor models [42]. In addition, previous studies shows leukocytes can be educated to upregulate the expression of Mer's ligand GAS6 in a Mer-dependent manner once they are recruited into tumor microenvironment [43], thus supporting a model in which Mer signaling induces GAS6 expression in tumor-associated macrophages, creating positive feed-forward signaling acting on tumor cells and tumor-associated macrophages through Mer [42]. In view of the abundant presence of tumor-associated macrophages and their pivotal role in tumorigenesis, it is of great significance to deepen our understanding of the biological effects of Mer in tumor stromal cells.

In sum, our data demonstrated that Mer RTK is frequently overexpressed in both tumoral and stromal compartments of NSCLC where it facilitates tumor progression by promoting the proliferation and migration of NSCLC cells via stimulating MAPK and FAK signaling pathway respectively. Of clinical significance, Mer expression dictates the sensitivity of NSCLC cells harboring EGFR mutation to EGFR-targeted agent erlotinib, providing a rationale to develop clinically translatable compounds inhibiting Mer as potential therapeutics to overcome the erlotinib resistance.

## MATERIALS AND METHODS

### Clinical samples and immunohistochemistry

The study protocol was approved by the Institution Review Board of the Second Military Medical University. TMAs with tumor tissues and matched peritumoral samples from 150 patients with NSCLC were purchased from Shanghai Biochip Company of China (Cat. HLug-Squ150/Sur-01, HLug-Ade150/Sur-01) which contained 150 consecutive cases of surgically resected NSCLC (surgery time July 2004 to November 2007). The detailed clinicopathologic characteristics of the patients are listed in Table 1. All patients were followed until July, 2012 with a median observation time of 60.5 months (range, 1–96 months). The time of the surgery was used to calculate the time to a given event. Overall survival (OS) was defined as the interval between surgery and date of death. The OS was censored at the last follow-up visit (July 31, 2012) for patients without death. The tumors were staged according to the International Union Against Cancer's tumor-node-metastasis (TNM) classification and histologically subtyped and graded according to the World Health Organization guidelines (third edition). Freshly harvested NSCLC samples (30 cases) were obtained from Department of Pathology, General Hospital of Chinese People's Armed Police Forces, Beijing, China. The detailed clinicopathologic characteristics of the patients are listed in Supplementary Table 1. The detailed procedure for the immunohistochemistry of Mer protein in the clinical samples is provided in the Supplementary Information.

All specimens were scored independently by two pathologists (LXW and HFW). An H-score was determined for each specimen by multiplying the percentage of positive tumor cells (0% to 100%) by the dominant staining intensity (0 = negative, 1 = weak, 2 = intermediate, and 3 = strong). Thus, resulting scores ranged from 0 to 300. Specimens with overall scores of 0 to 4, 5 to 100, 101 to 200, and 201 to 300 were classified as trace, low-level, intermediate-level, and high-level expression, respectively. When two scores for same sample from two pathologists were evaluated, the correlation coefficient was high (0.89), indicating that the agreement between the two pathologists was good. Based on this result we used the average scores of the two pathologists.

### Cell culture

The human bronchial epithelial cell line BEAS2B and NSCLC cell lines A549, H460, H1965, H2228, H1563, and H661 cells were all purchased from the American Type Culture Collection (Rockville, MD, USA) and PC9 NSCLC cells were obtained from Cell Bank of Chinese Academy of Medical Sciences (Beijing, China). These cells were all maintained in DMEM medium (Hyclone) supplemented with 10% fetal bovine serum (FBS; Hyclone), 100 units/l penicillin, and 100 μg/l streptomycin (Life Technologies). The BEAS2B and PC9 cells stably expressing control or Mer were cultured in similar medium additionally supplemented with 1 μg/ml puromycin.

### Transfection and infection

For lentiviral infection, human cDNA of Mer (NM_006343.2) was cloned into bicistronic pCDH-T2A-puro lentiviral vector (System Biosciences) between XbaI and BamHI restriction enzymatic site. 5 × 10^6^ 293T cells were transfected with 4 μg of pCDH empty vector or pCDH-Mer plasmids, and 3 μg of psPAX2 (Addgene), 1 μg of pMD2.G (Addgene), and 20 μl of Lipofactamine 2000 transfection reagent (Invitrogen). Forty-eight hours post-transfection, about 5 ml of conditioned medium containing viral particles was collected and filter sterilized. 500 μl of viral particles was used to infect 4 × 10^5^ BEAS2B or PC9 cells for 12 h after which viral conditioned medium were replaced with fresh medium. For stable expression, 1 μg/ml puromycin was used to select BEAS2B or PC9 cells stably expressing Mer or pCDH 72 h post-infection.

### Human GAS6 conditioned medium

The human GAS6 conditioned medium (CM) was prepared as described previously with minor modification [44]. Briefly, 5 μg of pCMV3-human GAS6 plasmid (Sino Biological Inc) was transfected into a 10-cm plate of 293T cells at 70% confluence using Lipofactamine 2000 (Invitrogen) in the presence of 10 μg/ml vitamin K1 (Hospira). After overnight incubation, the complete medium with 10% FBS was then changed into 0% serum in the presence of vitamin K1. About 24 h later, the GAS6 conditioned medium was collected. For mock-conditioned medium, blank pCMV3 plasmid was used.

### Clonogenic colony formation assay

The assay was performed as previously described [31]. Briefly, cells were plated in triplicate wells of 6-well dishes at a low density (150 cells/well) and cultured under normal conditions without perturbation for 10 days. In some assays, cells were cultured in the presence of kinase inhibitors (concentrations see below) with supplementing inhibitors every two days. Colonies were washed with PBS and stained with crystal violet (0.5% w/v in 25% methanol). Stained plates were rinsed in dH2O and allowed to dry at room temperature. The plates were photographed, and colonies were counted using ImageJ software.

### Proliferation and growth inhibition assays

For proliferation assay, BEAS2B cells stably expressing control or Mer (1 × 10^3^ cells per well) were cultured in the 6-well plates in medium containing full supplements. The cell proliferation was monitored every 3 days by cell counting with hemocytometer. For growth inhibition assay, cells (confluence 60–70%) were pretreated with Mer-specific inhibitor UNC569 (Selleck) or vehicle control (DMSO) for 12 h followed by incubation with serially diluted concentrations of erlotinib (Selleck) for 72 h. For experiments in PC9 cells, cells were directed with erlotinib without pretreatment procedure. At the termination of the experiment, 20 μl of MTT assay solution was added into 100 μl of medium containing cells and incubated for 2 h. The absorbance of each well was determined using a microplate reader (Molecular devices, Sunnyvale, CA) at 492 nm with reference wavelength at 630 nm. The percentage of cell survival was defined as the relative absorbance of untreated versus treated cells. All assays were performed in triplicate and repeated three times.

### Scratch/wound healing assay

BEAS2B cells stably expressing control or Mer (2 × 10^5^ cells per well) were plated on 6-well plates precoated with collagen type I (5 μg/cm^2^, BD Biosciences). Cells were incubated overnight yielding confluent monolayers for wounding. Wounds were made using a pipette tip and then cells were continued to culture in the presence of 20% (v/v) mock or GAS6 contained conditioned media. The photographs were taken immediately (0 h) and 24 and 48 h after wounding with a phase-contrast microscope (Leica Microsystems) equipped with a digital camera (Leica DFC300FX). Percentage of migration was analyzed by ImageJ software. MK2206 (4 μM), PF-562271 (1 μM), SB203580 (1 μM), or vehicle (DMSO) were added 1 h before wounding and maintained during migration. The experiments were carried out in triplicate and repeated at least three times.

### Western blotting analysis

Western blotting was done as we previously described [45] using the following primary antibodies: phosphorylated AKT (p-AKT, Ser473), phosphorylated ERK1/2 (p-ERK1/2, Thr202/Tyr204), phosphorylated FAK (p-FAK, Tyr397), phosphorylated EGFR (p-EGFR, Tyr1068), AKT, ERK1/2, FAK, PARP, and GAPDH from Cell Signaling Technology (Denvers, MA, USA) and phosphorylated Mer (p-Mer, Tyr749/753/754; ab14921) and Mer (ab52968) from Abcam (Cambridge, MA, USA). HRP-conjugated goat anti-rabbit secondary antibodies (Cell Signaling Technology) were used for enhanced chemiluminescence of western blots.

### Annexin V and 7-aminoactinomycin D staining

The determination of apoptotic cells was performed as we previously described [46]. Briefly, treated cells were washed twice in cold PBS and resuspended in Annexin V-binding buffer at a concentration of 3 × 10^6^ per ml. This suspension (100 μl) was stained with 5 μl of Annexin V-FITC and 5 μl 7-AAD. The cells were gently vortexed and incubated for 15 min at room temperature in the dark. After addition of 400 μl of binding buffer to each tube, cells were analyzed by flow cytometry immediately.

### Statistical analysis

Statistical analyses of immunohistochemistry data were performed by the Statistical Package for Social Sciences for Windows software (Windows version release 18.0; SPSS, Inc., Chicago, IL, USA). Descriptive statistics were calculated. Continuous variables were categorized: age at diagnosis was dichotomized at its median 63 years; Mer H-score was categorized according to convention. For clinical characteristics (age at diagnosis, histology and sex), statistical significance between different levels of each characteristic was assessed by log-rank test. On nodal status, differentiation, stage and categorical Mer H-score, statistical significance between different levels of each factor was assessed by log-rank test for trend as these variables are considered ordinal.

Statistical analyses of all other data were performed by GraphPad Prism (Version 5.04, GraphPad Software, Inc). Results are presented as mean ± S.D obtained from at least three independent experiments. Differences between groups were tested by two-tailed paired Student *t* test or one-way ANOVA followed by Tukey's multiple comparisons test, and *p* < 0.05 is considered as significant.

## SUPPLEMENTAL DATA


