# Supplementary material for: Mer receptor tyrosine kinase is frequently overexpressed in human non-small cell lung cancer, confirming resistance to erlotinib
Source: Oncotarget. 2015 Mar 16;6(11):9206–19. doi: 10.18632/oncotarget.3280 (PMC4496212; doi:10.18632/oncotarget.3280)
Supplement: Supplementary file 1 [file oncotarget-06-9206-s001.pdf]

SUPPLEMENTAL DATA

MATERIALS AND METHODS

Immunohistochemical staining of clinical tumor specimens

Deparaffinized sections were subjected to antigen retrieval using citrate buffer (Dako, S1699) in a humidified chamber. Allsubsequent steps were performed at room temperature. After rinsing in water and wash buffer, endogenous peroxidase blockade was achieved by incubating in 3% peroxide solution in water for 15 minutes. Nonspecific binding of antibodies and horseradish peroxidase (HRP) was reduced by 20 minute incubation in 3% bovine serum albumin in wash buffer. Rabbit monoclonal anti-Mer (ab52968 from Abcam) antibody was applied to stain sections at 1:50 in antibody diluent (Dako, S3022) for 1 hour in a humidified chamber following by the staining with the secondary antibody (Dako REAL™ EnVision™ Detection System, Peroxidase/DAB+ Rabbit/Mouse; Dako.K5007) for 40 minutes at room temperature. Visualization of staining was achieved by application of chromogen solution (DAB) as recommended by the manufacturer and slides were counterstained with hematoxylin (Dako, S3301) before mounting in xylene-based medium. As a negative control, we stained 4 Mer-positive NSCLC tissue samples using PBS instead of primary antibody where no specific staining was noted (Supplementary Fig. 1A), validating the specificity of anti-Mer antibody we used.

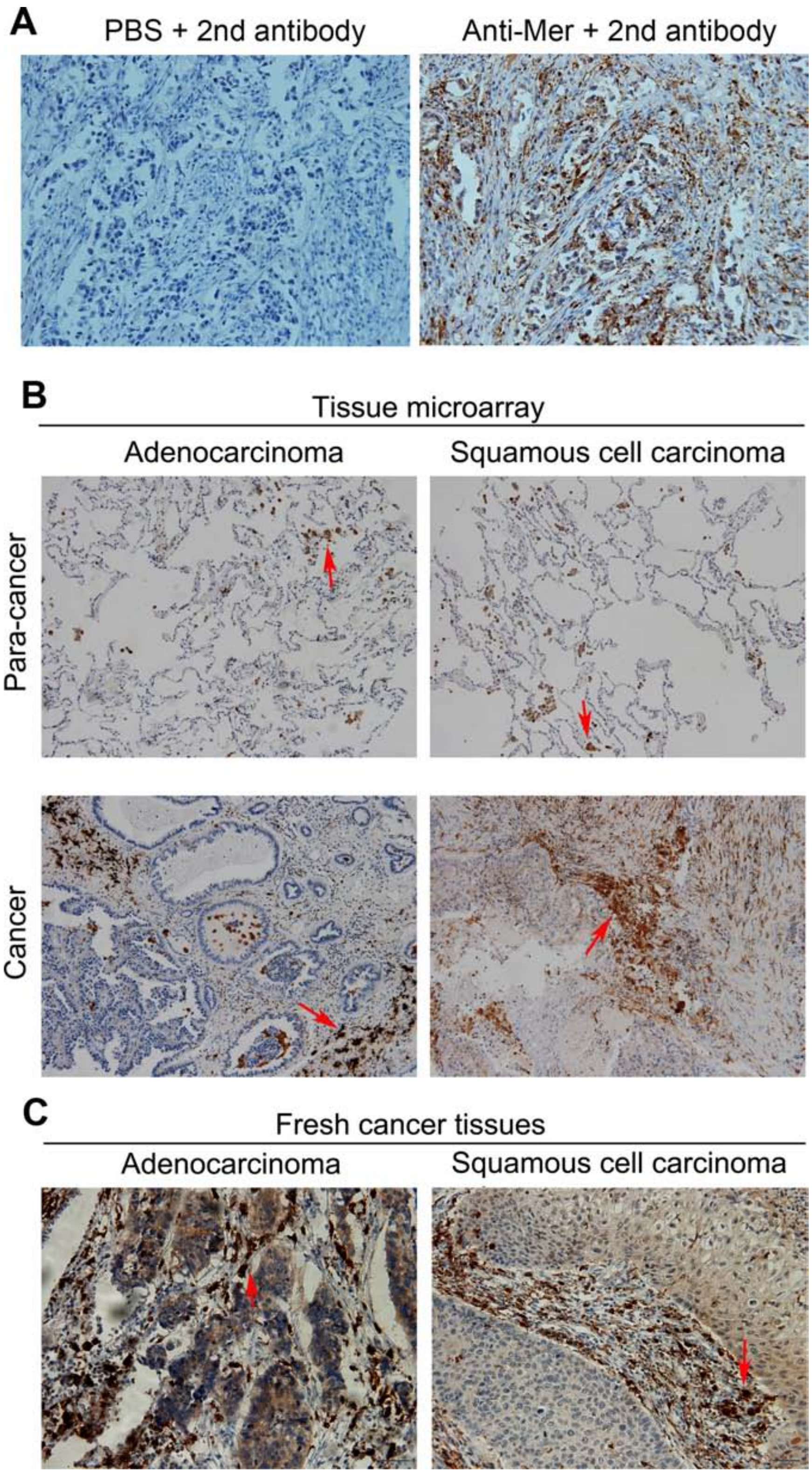

Supplementary Figure S1: A. Validation of anti-Mer antibody specificity. Four NSCLC tumor tissues were stained with primary anti-Mer antibody (right graph) or PBS (left graph) followed by HRP-labelled secondary (2nd) antibody. The representative IHC pictures from one sample were shown (100 × magnification). **B and C.** Mer RTK exhibits a pronounced expression on stromal cells with macrophage morphology (arrows) in NSCLC of TAM and fresh harvested tissues. The pictures from TMA were shown in 100 × magnification and scale bar in the pictures from fresh cancer tissues represents 50 μM.

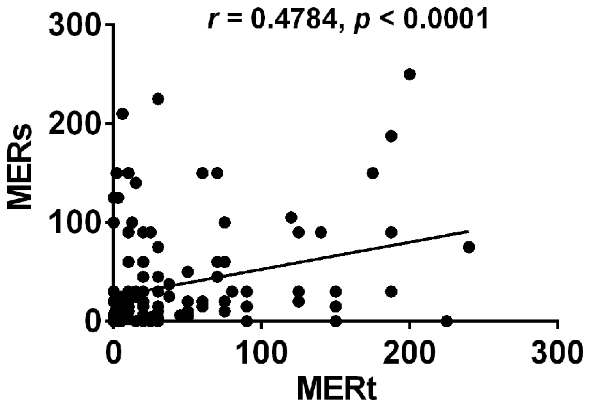

Supplementary Figure S2: The correlation of expression level between tumoral Mer and stroma Mer was evaluated by Pearson correlation analysis.

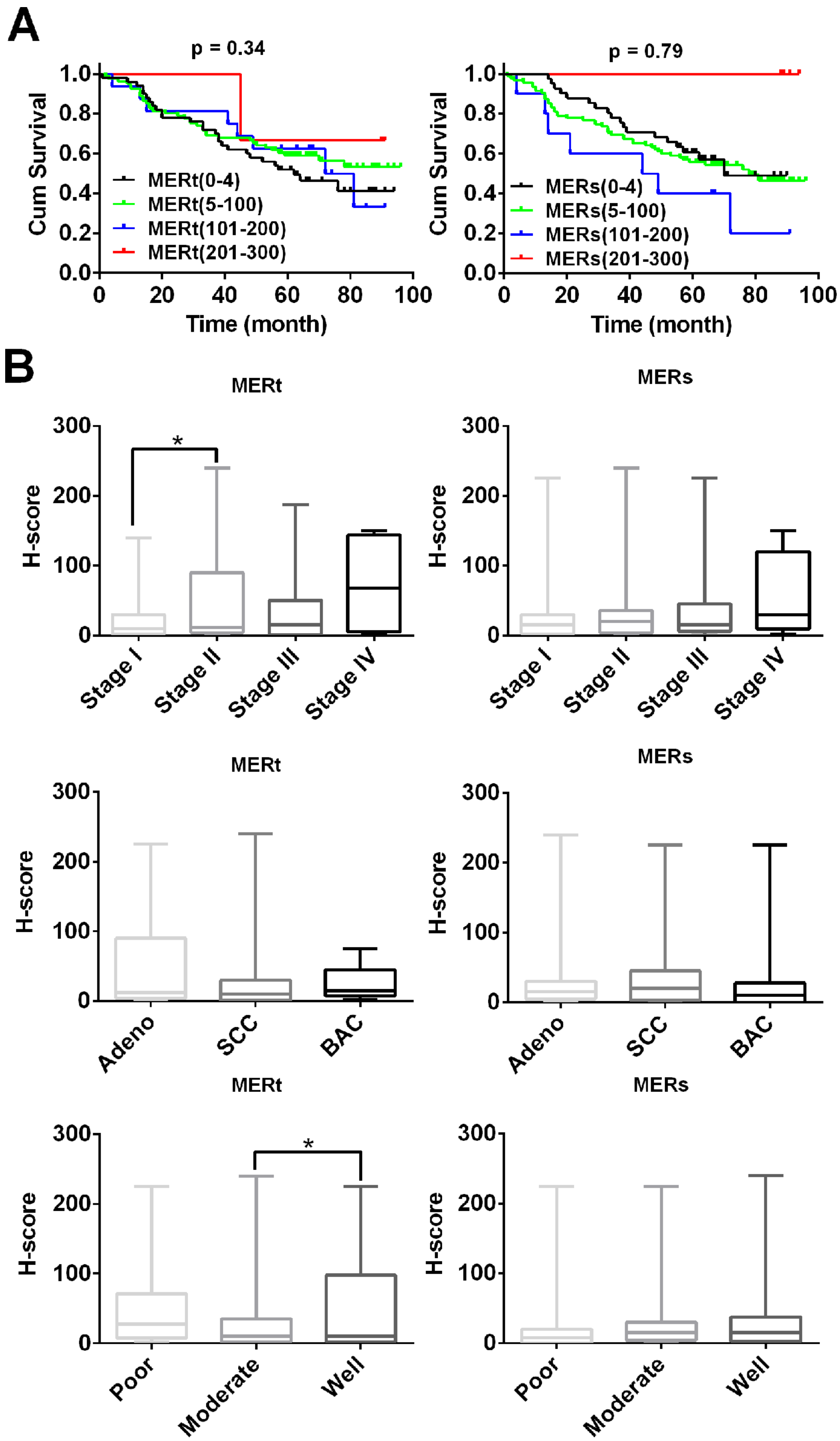

Supplementary Figure S3: Mer expression in tumoral and stromal compartments was not associated with tumor stage, histology and differentiation. A. Kaplan–Meier curves for overall survival of 150 NSLCC patients according to the Mer expression level in tumoral and stromal compartments over a median 60.5-month follow up period after initial surgery. **B.** Mer expression in tumoral and stromal compartments were compared according to tumor TNM stage, histology and differentiation. *$p < 0.05$, Log-rank test (for A) or ANOVA followed by Tukey's multiple comparisons test (for B).

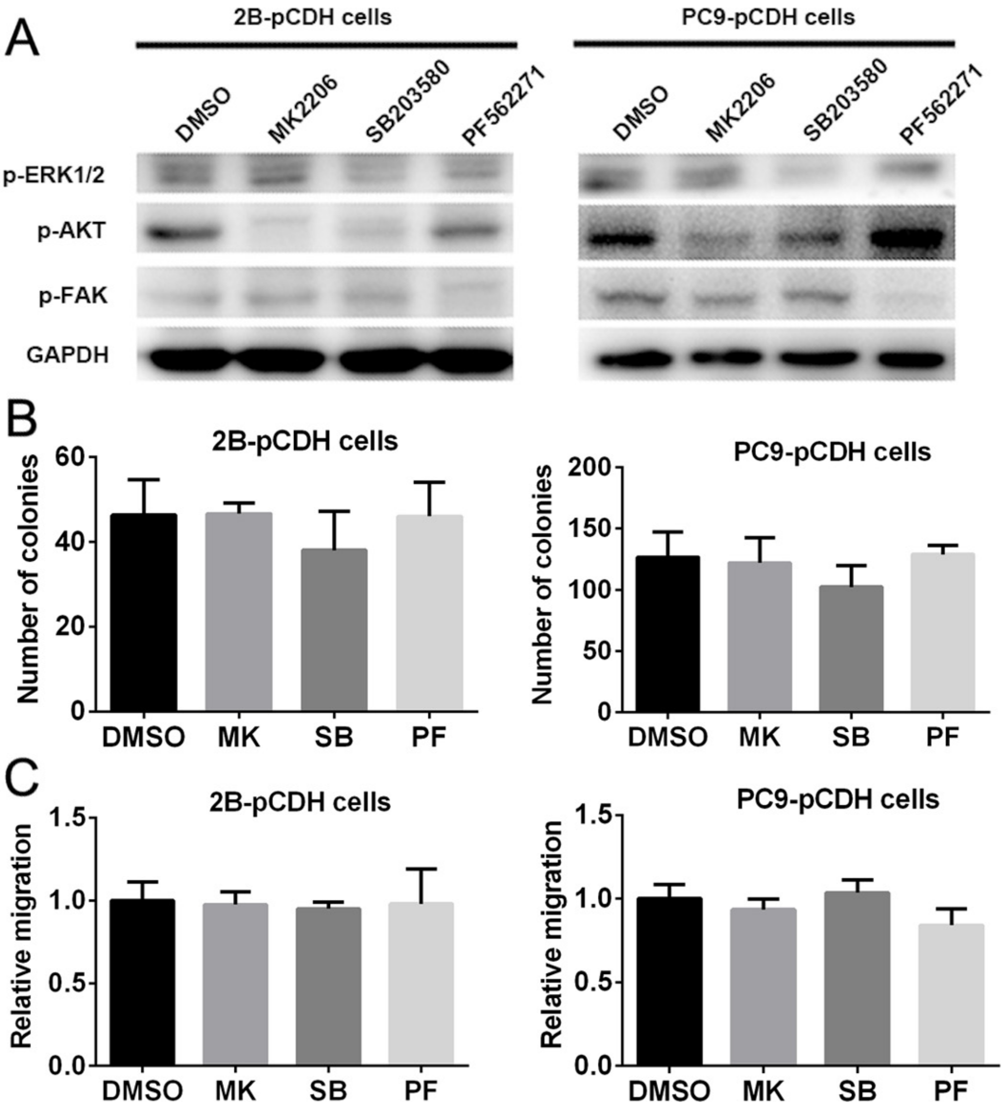

Supplementary Figure S4: A. blockade of signaling pathway activity downstream of Mer activation in control 2B-pCDH or PC9-pCDH cells treated with the corresponding signal inhibitors. Blots representative of two independent experiments were shown. **B.** Clonogenic colony formation from control 2B-pCDH or PC9-pCDH cells cultured in the presence of the corresponding inhibitors for MAPK (SB203580, 1 μM), FAK (PF-562271, 1 μM)) and AKT (MK2206, 4 μM)) signals with DMSO treatment as controls. **C.** Relative cell migration of control 2B-pCDH or PC9-pCDH cells 48 h after wounding in the presence of the corresponding inhibitors for AKT, MAPK and FAK signals with DMSO treatment as controls. Data are expressed as mean ± SD of triplicates and representative of two independent experiments.

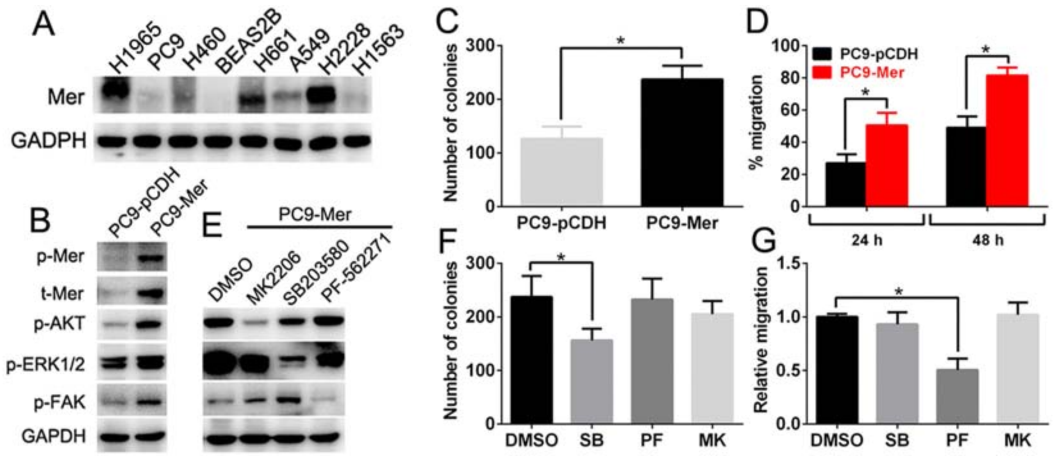

Supplementary Figure S5: Mer overexpression promotes the proliferation and migration of NSCLC cells. A. Mer expression in BEAS2B normal lung epithelial cells and 7 NSCLC cells was determined by western blotting. **B.** Mer-related signals were evaluated by western blotting using antibodies specific for total (t) and phosphorylated (p) proteins in PC9 cells stably expressing control (PC9-pCDH) or Mer (PC9-Mer) cultured in the presence of 20% (v/v) mock or GAS6 contained CM. **C.** PC9 cells stably expressing control (PC9-pCDH) or Mer (PC9-Mer) were plated in 6-well plates and cultured for 10 days and colony numbers were determined at the end of experiments. **D.** PC9 cells stably expressing control (PC9-pCDH) or Mer (PC9-Mer) were plated on 6-well plates where wounds were made using a pipette tip and cell migration into wounds were monitored at 24 and 48 h after wounding in the presence of 20% (v/v) mock or GAS6 contained CM. **E.** Blockade of signaling pathway activity downstream of Mer activation in PC9-Mer cells treated with the corresponding signal inhibitors. **F.** Clonogenic colony formation from PC9-Mer cells cultured in the presence of the corresponding inhibitors for MAPK (SB203580, 1 μM), FAK (PF-562271, 1 μM)) and AKT (MK2206, 4 μM)) signals with DMSO treatment as controls. **G.** Relative cell migration of PC9-Mer cells 48 h after wounding in the presence of the corresponding inhibitors for AKT, MAPK and FAK signals with DMSO treatment as controls. Blots representative of three independent experiments were shown. All data are expressed as mean ± SD of triplicates and representative of three independent experiments. *$p < 0.05$, paired student t test (for C and D) or ANOVA followed by Tukey's multiple comparisons test (for F and G).

Whole Genome RVista

Genomic coordinates and distance from transcription start of hits for factor P53

Chrom	Start	End	Strand	Relative Start	Relative End
chr2	112653759	112653778	-	-2432	-2413
chr2	112653769	112653788	+	-2422	-2403
chr2	112653777	112653796	+	-2414	-2395
chr2	112653777	112653796	-	-2414	-2395
chr2	112653867	112653886	+	-2324	-2305
chr2	112653868	112653887	+	-2323	-2304
chr2	112654261	112654280	-	-1930	-1911
chr2	112654266	112654285	+	-1925	-1906
chr2	112654891	112654910	+	-1300	-1281
chr2	112656045	112656064	+	-146	-127
chr2	112656049	112656068	-	-142	-123
chr2	112656055	112656074	-	-136	-117

Supplementary Figure S6: Prediction of p53 binding site on the promoter region of human *Mer* tyrosine kinase gene by online tool (Whole Genome RVista: http://genome.lbl.gov/cgi-bin/WGRVistaInputCommon.pl).

Supplementary Table S1. The clinical characteristics of 30 NSCLC patients

	***Patients* N (%)**
Age at diagnosis	
≤ 60 years	14 (47)
> 60 years	16 (53)
Gender	
Male	18 (60)
Female	12 (40)
Histology	
Adeno	17 (57)
SCC	13 (43)
Stage	
I	10 (33)
II	8 (27)
III	12 (40)
Differentiation	
Poor	4 (13)
Moderate	20 (67)
Well	6 (20)
MERt H-score	
0–4	9 (30)
5–100	13 (43)
101–200	5 (17)
201–300	3 (10)
MERs H-score	
0–4	8 (23)
5–100	11 (37)
101–200	8 (23)
201–300	2 (7)

Abbreviations: Adeno, adenocarcinoma; SCC, squamous cell carcinoma; MERt, MER in tumor; MERs, MER in stroma.

Supplementary Table S2. Summary of tumor formation in nude mice

Cells	*Number of cells injected*	*Number of mice injected*	*Tumor n (%)*
BEAS2B-Con	5×10^6	2	0 (0)
	1×10^7	3	0 (0)
BEAS2B-Mer	5×10^6	3	0 (0)
	1×10^7	4	0 (0)
BEAS2B-EML4-ALK	5×10^6	5	5 (100)
A549	5×10^6	1	1 (100)
	1×10^7	1	1 (100)

Female athymic nude mice (5–6 weeks old; Weitonglihua Biotechnology, Beijing, China) were maintained and treated under specific pathogen-free conditions. Mice were injected in the left flank area subcutaneously (s.c.) with 5×10^6 or 1×10^7 indicated cells in 100 μl of PBS. Mice were monitored for tumor formation twice weekly. BEAS2B-*EML4-ALK* are BEAS2B cells stably expressing a *EML4-ALK* transforming oncogene by lentivirus-mediated transduction (obtained from Prof. Hiroyuki Mano, Japan Science and Technology Agency, Japan; Ref 20)
